# Effect of Institution and COVID-19 on Access to Adult Arthroplasty Surgery

**DOI:** 10.1016/j.artd.2022.01.027

**Published:** 2022-01-24

**Authors:** Kylen K.J. Soriano, Paul Toogood

**Affiliations:** Department of Orthopaedic Surgery, University of California-San Francisco, San Francisco, CA, USA

**Keywords:** Arthroplasty, Wait time, COVID, Insurance, Disparity, Safety-net

## Abstract

**Background:**

Although insurance status is important to patients’ ability to access care, it varies significantly by race, age, and socioeconomic status. Novel coronavirus disease 2019 (COVID-19) negatively impacted access to care, while simultaneously widening pre-existing health-care disparities. The purpose of the present study was to document this phenomena within orthopedics.

**Methods:**

Patients undergoing hip or knee arthroplasty at two medical centers in San Francisco, California, were evaluated. One cohort came from the University of California San Francisco (UCSF), a tertiary center, and the other from Zuckerberg San Francisco General Hospital (ZSFGH), a safety-net hospital. Patients who underwent arthroplasty before the pandemic (March 2020) and those after pandemic declaration were evaluated. Patient demographics, surgical wait times, and operative volumes were compared.

**Results:**

Two-hundred sixty-nine (pre-COVID, 184; post-COVID, 85) cases at UCSF and 63 (pre-COVID, 47; post-COVID, 16) cases at ZSFGH met inclusion criteria. Patients at ZSFGH had a significantly higher body mass index, were more often racial minorities, and were less likely to speak English. Patients at ZSFGH were less likely to have private insurance. A comparison of case volumes showed a larger decrease at ZSFGH than at UCSF after COVID. Wait times between the two sites before and after COVID showed a larger increase in wait times at ZSFGH. Notably, wait times at ZSFGH before COVID were more than double the wait times at UCSF after COVID.

**Conclusions:**

COVID-19 worsened access to primary hip and knee arthroplasties at two academic medical centers in San Francisco. The pandemic also worsened pre-existing disparities. Racial minorities, non-English speakers, and those with nonprivate insurance were affected the most.

## Introduction

Insurance status is a well-known predictor of patients’ ability to access medical care in the United States [1]. While the Affordable Care Act expanded coverage for many Americans, there are still large disparities [[2], [3], [4], [5]]. Lower rates of provider reimbursement, onerous paperwork, and increasing clinical complexity and comorbidities associated with Medicaid-insured patients have all been hypothesized to contribute [6]. These disparities in access have been well documented not just in primary care but also specifically in elective and nonelective orthopedic care [[7], [8], [9], [10], [11]].

More recently, studies have focused on the novel coronavirus disease 2019 (COVID-19) pandemic and its effects on access to medical care [12]. By the time COVID-19 was declared a pandemic by the World Health Organization [13], recommendations had been made by the American College of Surgeons [14], the Centers for Disease Control [15], and the American Academy of Orthopedic Surgeons [16] to postpone all elective surgical cases. As a result, rates of elective procedures, such as total knee arthroplasty (TKA) and total hip arthroplasty (THA), decreased dramatically across the United States beginning in March 2020. While resource allocation and restrictions on elective procedures affected all institutions nationwide, it potentially affected underinsured populations more substantially [17,18].

The aim of the present study is thus to evaluate the effect of the COVID-19 pandemic on access to orthopedic care using primary hip and knee arthroplasty as a demonstrative, common, elective surgical procedure. We hypothesize that access to care worsened after the pandemic for all patients undergoing elective THA and TKA and that patients seeking care at a public institution, which provides an unbalanced amount of care to the underinsured, were disproportionately impacted.

## Material and methods

### Patient selection and data collection

After receiving institutional review board approval, a retrospective review was performed of adult patients who had undergone primary THA or TKA at two academic medical centers in San Francisco, California. One cohort was obtained from the University of California San Francisco (UCSF), a tertiary referral center for the region, and the other cohort from Zuckerberg San Francisco General Hospital (ZSFGH), the public hospital for the city and county of San Francisco. From each institution, data from two time periods were collected, one just before the declaration of the pandemic (pre-COVID) and one just after (post-COVID). At UCSF, the pre-COVID group consisted of adult patients undergoing hip and knee arthroplasty (Current Procedural Terminology codes 27130, 27134, 27137, 27138, 27446, 27447, 27486, 27487) from December 1, 2019, to February 29, 2020, and the post-COVID group consisted of such patients from March 1, 2020, to May 31, 2020. At ZSFGH, owing to the much lower baseline volume of cases, these time frames were expanded (September 1, 2019, to February 29, 2020, [pre-COVID] and March 1, 2020, to August 31, 2020, [post-COVID]) to allow for larger numbers for comparison.

After evaluating individual records, patients were excluded (Table 1) if the procedures had been performed for revision, were resection arthroplasties, were associated with hardware removal, or performed for fracture. Patients were also excluded if surgery had been delayed because of patient preference, bilateral staging, or substance abuse screening, all of which were felt to artificially influence wait times.

**Table 1:** Exclusion criteria.

Case data	UCSF	ZSFGH
Total arthroplasty (hip and knee)	375	111
Total exclusions	106	48
Revision	78	20
Resection arthroplasty/hardware removal	0	6
Fracture	6	3
Patient preference	17	14
Bilateral staging	5	0
Active substance use	0	5
Final cohort	269	63

Values are presented as no. of patients unless otherwise specified.

With pre- and post-COVID groups obtained from both institutions, medical records were evaluated to extract patient demographics and insurance status (Table 2). Surgical wait times were then calculated for each group; this was defined as the time from first documented recommendation for surgery by a member of the arthroplasty service (attending physician, physician assistant, or nurse practitioner) to actual operative date. Surgical volume was also tabulated and compared between the two institutions during the study time periods.

**Table 2:** Demographics for UCSF and ZSFGH cohorts.^a^

Demographics	UCSF	ZSFGH	*P* value^b^
Cohort, pre-COVID/COVID	184/85	47/16	**.417**
Sex, male/female	113/156	21/42	**.263**
Age, mean ± SD, y	63.6 ± 11.5	63.3 ± 7.4	**.818**
Body mass index, mean ± SD, kg/m^2^	28.5 ± 5.3	33.4 ± 6.0	<.001
White, non-Hispanic (%)	68.8%	17.5%	<.001
English preferred language (%)	94.8%	60.3%	<.001
Insurance status			
Private insurance, non-Medicare (%)	65.4%	1.7%	<.001
Medicare as primary insurance (%)	26.4%	49.2%	<.001
Multiple private insurers (%)	49.4%	0%	<.001
Has public insurance (%)	18.2%	98.4%	<.001
Only public insurance (%)	8.2%	47.6%	<.001

Bold values indicate statistical significance.

a Values are presented as no. of patients unless otherwise specified.

b Student unpaired-samples t-test for mean values and chi-square test for categorical values.

### Statistical analysis

Unpaired student’s t-tests were used to evaluate for significance between surgical wait times. A chi-square test was used to assess significance difference between categorical variables in each cohort (sex, race, insurance status). A *P* value of <0.05 was considered statistically significant for all calculations. All statistical computations were conducted in RStudio (version 1.3.959; RStudio: Integrated Development Environment for R).

## Results

During the study time periods, a total of 375 and 111 cases, as determined by Current Procedural Terminology codes, were performed at UCSF and ZSFGH, respectively. After review, 106 cases were excluded from UCSF, and 20 cases were excluded from ZSFGH (Table 1), leaving a total of 269 patients from UCSF and 63 at ZSFGH for analysis.

Demographic findings for the two cohorts are provided in Table 2. There was no significant difference between the groups in sex or age distribution. Patients at ZSFGH had a significantly higher average BMI, were less likely to be White, and less likely to use English as their preferred language. Patients at ZSFGH were significantly less likely to have private insurance coverage and significantly more likely to have public insurance coverage, such as San Francisco Health Plan or MediCal.

There were 184 patients in the UCSF pre-COVID cohort and 85 patients in the UCSF post-COVID cohort; this represents a case volume decrease of 53.8%. There were 47 patients in the ZSFGH pre-COVID cohort and 16 patients in the ZSFGH post-COVID cohort; this represents a case volume decrease of 66.0% (Table 3). The mean surgical wait times at UCSF were 115.5 ± 5.4 days for the pre-COVID cohort and 132.1 ± 6.4 days for the post-COVID cohort; this represents a mean increase of 16.6 days (14.4% increase). The mean surgical wait times at ZSFGH were 291.8 ± 14.9 days in the pre-COVID cohort and 344.8 ± 29.6 days in the post-COVID cohort; this represents a mean increase of 53 days (18.1% increase) (Table 4).

**Table 3:** Case volume UCSF vs ZSFGH.

Case volume	UCSF cohort	ZSFGH cohort	*P* value
Pre-COVID	184	47	
COVID	85	16	
Difference (N)	−99	−31	
Difference (%)	−53.8%	−66.0%	**.417**

Bold values indicate statistical significance.

**Table 4:** Wait times UCSF vs ZSFGH.

Surgical wait time (mean ± SE)	**UCSF cohort**	**ZSFGH cohort**	***P*****value**
Pre-COVID (d)	115.5 ± 5.4	291.8 ± 14.9	<.001
COVID (d)	132.1 ± 6.4	344.8 ± 29.6	<.001
*P* value^a^	**.034**	**.045**	
Difference (d)	+16.6	+53.0	
Difference (%)	+14.4%	+18.1%	

Bold values indicate statistical significance.

a Student unpaired-samples t-test for mean values and chi-square test for categorical values.

## Discussion

Insurance status is a well-documented predictor of access to orthopedic surgical care in the United States. Prior authors, before the pandemic, documented increased surgical wait times for knee arthroplasty, ankle fracture fixation, anterior cruciate ligament reconstruction, and shoulder stabilization procedures in patients who lacked insurance or were underinsured [[7], [8], [9], [10], [11]]. These mirror the findings of the present study. Patients who sought care at the public hospital (ZSFGH) were far less likely to have private insurance and had significantly longer mean wait times for hip and knee arthroplasty than those seeking care at the private, academic center (UCSF) even before the COVID-19 pandemic. Also, similar to prior research, patients seeking care at the public, safety-net hospital were more often non-White and/or non-English speaking [[19], [20], [21], [22], [23], [24]].

The COVID-19 pandemic necessitated a temporary shift of resources away from elective orthopedic surgical care [[21], [22], [23], [24]]. Prior authors have documented up to 80% of US orthopedic surgeons experiencing a decline in surgical volume after the onset of the pandemic [25]. Similarly, in Europe, in April of 2020, it has been estimated that 92.6% of joint replacements were canceled [26]. Again, the findings of the present study are consistent with prior reports, with both institutions having clear reductions in surgical volume and increases in wait times immediately after the onset of the pandemic.

To the senior author’s knowledge, this is the first study in the orthopedic literature to evaluate the compounding effects of pre-existing underinsurance status and the COVID-19 pandemic on access to elective orthopedic surgical care. As would be predicted, pre-existing disparities only worsened, with case volumes falling more and surgical wait times ballooning further at the safety-net hospital (ZSFGH) relative to the private, academic center (UCSF). Perhaps of particular note, the pre-COVID wait times at ZSFGH were more than double the post-COVID wait times at UCSF. The exact reasons for the disparate baseline and post-COVID access to TKA and THA at the two institutions are complex and not assessed in this study. The baseline reserve of the institutions and the socioeconomic status of the patients are doubtlessly different and likely largely explain the findings. However, the authors would suggest that all the *direct* causes not exposed in this study can likely be understood as ultimately *indirectly* caused by the existence of systematic policies, laws, and practices that provide differential access to patients seeking care at pubic institutions. The present study thus further highlights the importance of reform measures aimed at reducing existing institutionalized racial disparities.

This analysis is not without limitations. The time periods compared between the two institutions was not the same. The comparison was performed in this manner to allow for a more meaningful number of patients to compare at ZSFGH. Specifically, when the cohorts of patients gathered from ZSFGH included only the 3 months immediately preceding and following the declaration of the pandemic, only 17 and 4 patients met inclusion criteria for pre- and post-COVID groups, respectively. By expanding the time frame to 6 months before and after pandemic declaration, more meaningful numbers of patients were included (47 pre-COVID and 16 post-COVID). However, restrictions on the use of operating rooms were not static during this period and, thus, could have affected the results. As restrictions tended to lessen over time (eg, at ZSFGH, 4 cases were performed during the first 3 months after pandemic declaration compared with 12 performed during months 4-6), it seems likely that the mean wait time and case volume in the ZSFGH post-COVID cohort likely benefitted from the longer time interval evaluated, thus not reducing the validity of the results. While the UCSF cohorts could also have been expanded to a longer 6-month time frame, the authors did not think this was required to obtain sufficient numbers for comparison and felt UCSF more quickly increased case volumes for elective surgery, including in May through August of 2020, which would then only further serve to strengthen the already compelling findings presented. In addition, while exclusion criteria were technically the same, substance abuse screening delays are an unusual feature of practice at ZSFGH (and not UCSF) and, thus, deserve special mention. As much as one-quarter of patients presenting for arthroplasty at ZSFGH are actively using illicit substances or have done so within the last 12 months. This was found to correlate highly with postoperative complications at this institution, and so a previously published “sobriety pathway” was developed and has been in place for 2 decades [27]. This requires patients to have a documented period of sobriety for 12 months before proceeding with arthroplasty. This obviously intentionally elongates surgical wait times in this population at this hospital, and they were thus excluded from analysis. In addition, all surgeries were performed at two institutions, which limits the generalizability of the results to other institutions and geographic locations. Finally, only wait times and case volumes were used to measure access to surgical care. Other studies have also assessed the number of appointments before surgery, complication rates, and long-term outcomes as at least equally valid measures.

## Conclusion

The COVID-19 pandemic acutely worsened access to surgical care worldwide, including access to primary hip and knee arthroplasty at two medical centers in San Francisco, California. However, the pandemic did not affect the populations treated at these hospitals equally, instead worsening pre-existing disparities as assessed by wait times and surgical volume. Racial minorities, non-English speakers, and those with nonprivate insurance were affected most, widening the large gaps that preceded the pandemic.

## Conflicts of interest

The authors declare the following financial interests/personal relationships which may be considered as potential competing interests: P. Toogood is a AAOS OITE member.

For full disclosure statements refer to https://doi.org/10.1016/j.artd.2022.01.027.

## Appendix A Supplementary data

Conflict of Interest Statement for Soriano

Conflict of Interest Statement for Toogood
